# Topics of the Month

**Published:** 1897-05

**Authors:** 


### TOPICS OF THE MONTH.

The College of Agriculture of Cornell University has undertaken to assist, free of expense, all teachers who wish to introduce nature-study into their schools. The idea of seeing familiar things in a new light is a valuable factor in education. For instance, how many people can explain, so that a child can understand, why water puts out fire, why some young squash plants bring their shells out of the ground on their backs and others do not; or show the difference between a leaf-bud and a fruit-bud of the apple; or tell from whence all the house flies come? The world is full of such common things, about which people do not inquire. Yet, such subjects can be made very interesting to children and they can be taken up in the schools, not as an added recitation, but as a rest exercise once or twice each week to relieve the monotony of the school-room and later be made the theme for a language exercise. Here are two important faculties that may be brought into exercise,—accurate observation and the power of expressing definitely what is seen. Parents and teachers interested in this work are asked to send their address for more detailed information to the chief clerk, College of Agriculture, Ithaca, N. Y.

---

CONSUMPTION caused 13,257 deaths in this state last year, a greater number than occurred from any other single cause. According to the annual report of the state board of health, there were 200 deaths in each 100,000 of the population, and it further shows that the total number of deaths for the year was 124,000, making the death-rate for the state 18.50 in each 1,000 of population. From the foregoing figures it ought not to be difficult to make people understand how important it is to adopt stringent preventive measures with reference to the spread of consumption. Spitting in public places is a filthy habit, but it is also a dangerous one, as the germs of infectious diseases like consumption are thereby spread to an amazing extent. Particles of dried sputum are taken up by the air and wafted hither and yon, easily finding a soil favorable to their propagation and growth. By all means let us have an ordinance, and that quickly, to suppress this dangerous custom.

---

THE health department of Buffalo has scored an important victory within the past month in the conviction of two physicians for violation of the ordinance relating to reporting contagious diseases. If the public is to be protected against the spread of contagious disease, stringent measures are necessary, requiring that any such shall be reported promptly to the health department. Failing to comply with such important laws, as in the present instances, should be met with swift conviction and sudden punishment. This is a feature of public health protection that admits of no middle-grounded action, and the city health commissioner is entitled to great credit in securing convictions in the cases referred to in the face of many discouragements and in spite of an adverse legal opinion.

---

THE bubonic plague at Bombay is reported to be rapidly waning. At the end of December it reached its climax with 1400 or more deaths a week. It has now fallen to less than one-third of that mortality. The city of Bombay is undergoing a thorough sanitary renovation; 130,000 dwelling houses were condemned, 109 ordered rebuilt, tiles removed from 1,027, floors dug up in 492, several hundred were lime-washed, 299 ordered vacated and three destroyed by fire. Indeed, the infected quarter is being dealt with in a fashion beyond the criticism of the most advanced sanitarian. It is doubtful if a more satisfactory sanitary achievement has ever been made before, and it reflects the highest credit on the administrators of sanitary law in that plague-stricken city.

---

The Murphy button has received recent further strong foreign endorsement. A press dispatch to the New York *Tribune*, dated Berlin, April 24, 1897, says: "At the Surgical Congress, in session here, Professor Marwedel, of Heidelberg, strongly recommended the general adoption of the American Murphy button in operations of the intestines and cited ninety-seven successful cases in his own practice."

---

The Philadelphia Polyclinic and College for graduates in medicine has set apart a room in the Polyclinic hospital for the free care of physicians who may become ill. The conditions which surround admission to its benefits are: that the applicant must be a member of the mutual aid association of the Philadelphia county medical society; that the officers of the association must first certify that the applicant's circumstances require such concession in regard to hospital charges; that the rules of the hospital in other respects in regard to the admission and duration of stay of patients be observed; and that the room may be used for other patients at regular rates when it is not occupied by a member of the association named.

---

The ordinance against spitting in street cars is being enforced in Philadelphia, where lately a passenger has been arrested and fined $5 for persistently violating it, though requested by the conductor several times to desist. In other near cities, notably Rochester and Scranton, the subject is engaging attention and means are being discussed or adopted to reform the evil of spitting in public places. In Rochester several persons have been fined for spitting on the sidewalks. In Scranton the newspapers are urging the passing of an ordinance which shall prevent the further exhibition of this filthy and dangerous practice. We hope to see a similar wholesome ordinance in Buffalo and its equally thorough enforcement. Too much attention to cleanliness cannot be paid in the street cars, where all sorts and conditions of people ride every day in the year.

